# Facile Synthesis of Sn/Nitrogen-Doped Reduced Graphene Oxide Nanocomposites with Superb Lithium Storage Properties

**DOI:** 10.3390/nano9081084

**Published:** 2019-07-28

**Authors:** Quan Sun, Ying Huang, Shi Wu, Zhonghui Gao, Hang Liu, Pei Hu, Long Qie

**Affiliations:** 1 Institute of New Energy for Vehicles, School of Materials Science and Engineering, Tongji University, Shanghai 201804, China; 2 Hubei Wanrun New Energy Technology Development Co., Ltd., Shiyan 442500, China; 3 State Key Laboratory of Material Processing and Die & Mould Technology, School of Materials Science and Engineering, Huazhong University of Science and Technology, Wuhan 430074, China

**Keywords:** Sn anode, N doping, graphene nanocomposites, rate performance, lithium-ion batteries

## Abstract

Sn/Nitrogen-doped reduced graphene oxide (Sn@N-G) composites have been successfully synthesized via a facile method for lithium-ion batteries. Compared with the Sn or Sn/graphene anodes, the Sn@N-G anode exhibits a superb rate capability of 535 mAh g^−1^ at 2C and cycling stability up to 300 cycles at 0.5C. The improved lithium-storage performance of Sn@N-G anode could be ascribed to the effective graphene wrapping, which accommodates the large volume change of Sn during the charge–discharge process, while the nitrogen doping increases the electronic conductivity of graphene, as well as provides a large number of active sites as reservoirs for Li^+^ storage.

## 1. Introduction

To meet the increasing demand for high-energy-density lithium-ion batteries (LIBs), it is critical to develop high-capacity anode and cathode materials with long-term cycling stability and fast charging–discharging capability [1,2]. On the anode side, many high-capacity candidates, such as silicon-based anodes, transition metal oxides, lithium metal, etc., have been investigated as substitutes for the commercial graphite anodes, with low theoretical capacity of only 372 mAh g^−1^ and limited rate performance. However, the commercialization of these materials is impeded by their limited cycling stability, low initial Coulombic efficiency, or complicated synthesis processes. For instance, Si-based composite anode material is being trialed for commercialization but the Si content is limited to avoid the large volume change (300%) of the electrodes, while for the Li metal anode, the safety concerns of Li dendrites still hinder its application, although great progress has been achieved in recent years. Thus, the development of anode materials with a high specific capacity and good cycling stability, as well as easy synthesis, is still challenging yet desirable for LIBs.

Among those promising candidates, the Sn anode has attracted much attention due to its high theoretical capacity (993 mAh g^−1^ for Li_4.4_Sn), high electrical conductivity, low-cost, and eco-friendly nature [3,4,5,6,7]. However, Sn undergoes a dramatic volume change of up to 259% and is prone to pulverization upon multiple lithiation–delithiation cycles, which induces the continuous break of formation of the solid-electrolyte interface (SEI), and thereby results in fast capacity fading. To address the aforementioned drawbacks and improve the cycling stability for practical application, construct hybrid structures (including yolk-shell structures, core-shell structures, and carbon coating) consisting of nanosized Sn and various carbons, such as carbon nanotubes, carbon nanowires, etc., have been developed [8,9,10]. Ishihara et al. found that coating Si with carbon nanotubes improved the cycle stability of the Si anode [11]. Lee et al. observed that homogeneously dispersed Sn nanoparticles in a polymerized C60 matrix improved the electrochemical performance of the composites [12]. All of these studies suggest that the carbon matrix not only increases the electrical conductivity but also buffers the large mechanical strain during cycling, as well as maintains the stability of the solid electrolyte interface (SEI) films. 

Graphene, a novel two-dimensional (2D) graphitic carbon, has drawn special attention and is preferable to support alloy or metal oxides to improve the electrochemical performance of the electrode materials in various types of rechargeable battery systems due to its outstanding mechanical flexibility, excellent electrical conductivity, and high chemical stability [13,14,15]. Hence, many Sn–graphene hybrids, such as 3D Sn@graphene [16,17], graphene decorated with Sn nanoparticles [18,19], and sandwich-like graphene-supported hybrids, have been developed to enhance the electrochemical performances of the hybrids [20,21,22,23]. In addition, previous reports indicated that the introduction of heteroatoms into the carbon lattices could improve their electrical conductivity, electrolyte wettability, and provide active reaction sites, eventually improving the lithium storage properties of grapheme [24,25,26]. However, construction of Sn and N-graphene hybrid anodes still needs to face the following obstacles: (i) the graphene has a tendency to aggregate or restack owing to the strong van der Waals forces existing between graphene layers, resulting in a seriously reduced active surface area; (ii) the N-doped graphene materials are derived from high temperature heat treatment of nitrogen sources and graphene oxide, which causes severe aggregation and poor structural stability; (iii) the Sn nanoparticles in the graphene are out of efficient control due to their low melting point of 232 °C, which accelerates the evaporation of Sn into large beads at high sintering temperatures and segregates the graphene base. This phenomenon is similar to the lotus effect, in which water spilled on a lotus surface does not wet the surface, but simply rolls off. Therefore, it is highly urgent to construct Sn@N-G hybrids with unique structures and excellent mechanical properties that can prevent the aggregation of graphene and evaporation of Sn nanoparticles to achieve superior electrochemical performances.

Herein, we report a facile approach for the synthesis of N-doped rGO networks anchored with Sn nanoparticles (Sn@N-G) as superior LIB anodes. This strategy involves the initial hydrothermal reaction of graphene oxide (GO) solution with SnCl_2_ and melamine, and the subsequent carbonization process, in which melamine sponges can construct a 3D carbon network and prevent the evaporation of Sn, allowing high structural stability of graphene. This configuration of Sn@N-G provides many remarkable advantages, such as the flexible graphene, to accommodate the volume change of Sn nanoparticles, N-doping to provide amounts of active sites as reservoirs for Li^+^ storage, with a 3D N-doped rGO (N-G) network to increase the electric conductivity of the electrode. As a result, the as-synthesized Sn@N-G hybrids exhibit a high reversible specific capacity of 1226 mAh g^−1^ at 0.1C, a superior rate capability of 535 mAh g^−1^ at 2C, and an excellent cyclability up to 300 cycles with a capacity retention of 939 mAh g^−1^ at 0.5C.

## 2. Materials and Methods

The graphene oxide (GO) was synthesized by the modified hammer’s method. In a typical synthesis, 0.5 g of SnCl_2_·2H_2_O and 0.25 g of melamine foam were dissolved in 30 mL of GO solution (2 mg mL^−1^). After stirring for 15 min, the mixture was transferred into an autoclave and heated to 180 °C for 6 h. After being cooled down to room temperature, the product was collected and washed with water and alcohol three times. After drying for 12 h at 60 °C, the black powers were heated to 800 °C in a tube furnace with a ramp rate of 5 °C min^−1^ and then held for 2 h. The whole annealing process was carried out in an Ar atmosphere. After the furnace was completely cooled to room temperature, the Sn-N-G composite was obtained.

To prepare the electrode slurry, the as-prepared Sn@N-G composite was mixed with carbon nanotubes (CNTs), conductive carbon black (Super P), and 5 wt% poly(vinyl difluoride) (PVDF) in the N-methyl pyrrolidone (NMP) solution. The weight ratio of the mixture was 7:1:1:1. The obtained slurries were pasted onto a Cu foil (Φ14 mm) and then dried in the vacuum at 80 °C overnight. The mass loading of active material in each cell was determined to be ~2 mg cm^−2^. The CR2025-type coin cells were fabricated in a high-purity argon-filled Mbraun glovebox (Germany) with H_2_O and O_2_ concentrations lower than 0.1 ppm, using lithium metal as the counter or reference electrode, Celgard 2400 separator and 1 M LiPF_6_ in a mixture of ethylene carbonate (EC) and diethyl carbonate (DEC) (1:1 in volume) as the electrolyte. The galvanostatic charge and discharge experiments were performed on a Land battery tester in a temperature-controlled thermotank with a potential range of 0.002–3.0 V.

The morphology of Sn@N-G was investigated by Scanning electron microscopy (SEM) (FEI SIRION200, Hillsboro, OR, USA) and Transmission electron microscopy (TEM) FEI Tecnai G2 F30, Hillsboro, OR, USA). The structures of materials were detected by X-ray photoelectron spectroscopy (XRD) (PANalytical X’pert PRO-DY2198, Almelo, The Netherlands) and Raman (HORIBA LabRAM HR800, Paris, France) measurements(532 nm wavelength laser). Surface chemical compositions were measured by X-ray photoelectron spectroscopy (XPS) by using Al Kα radiation (Ulvac-Phi PHI5000 Versa Probe, Kanagawa, Japan).The content of Sn was determined using a TG thermogravimetric analyzer (PE Diamond, Waltham, MA, USA) under an air atmosphere with a heating rate of 10 °C min^−1^ from room temperature to 600 °C.

**Figure 1:**
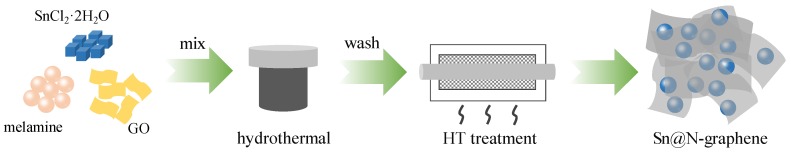
Schematic diagram of the synthesis of Sn@N-G composite.

The overall synthetic procedure for the Sn@N-G composite is illustrated in Figure 1. The procedure involves an initial hydrothermal process to form a 3D graphene-wrapped Sn precursor followed by high-temperature treatment in an inert atmosphere to reduce the graphene oxide precursor, as well as achieve nitrogen doping. Melamine was adopted as a nitrogen source due to its high nitrogen content, as reported previously [27]; the melamine SEM schematic is shown in Figure S1. Images of Sn@N-G composite particles are shown in Figure S2b, compared with Sn@G composite particles (Figure S2a).

## 3. Results and Discussion

**Figure 2:**
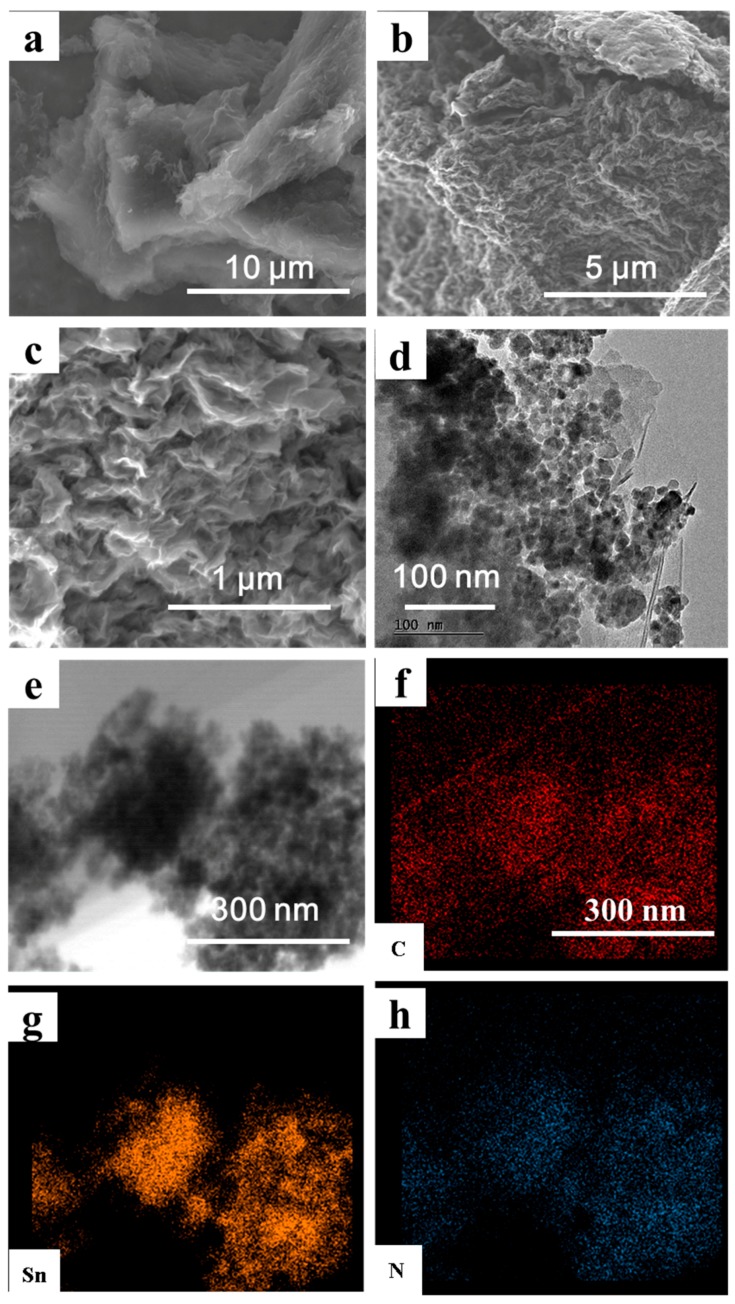
Characteristics of the Sn@N-G composite. (**a**–**c**) Scanning electron microscopy (SEM) images of the Sn@N-G composite; (**d**) Transmission electron microscopy (TEM) images of the Sn@N-G composite; (**e**–**h**) the mapping of Sn, N, and C elements.

The morphologies of Sn@N-G composites have been characterized by scanning electron microscopy (SEM), as shown in Figure 2a–c. The 2D sheet-like graphene layers with numerous folds at the edge can be obviously observed and the 2D sheets were stacked into a 3D matrix. However, the Sn nanoparticles are wrapped into the 3D graphene matrix. To further analyze the morphologically detailed information of Sn@N-G, TEM was applied, and the TEM images are shown in Figure 2d. In details, the graphene sheet exhibits a porous structure, along with numerous folds. Compared with the pure Sn particles, the graphene sheet matrix not only prevents the volume change but also construct a 3D conductive network for the electron transport, which is helpful in improving the electrochemical performance of the Sn@N-G composite electrode. The 3D graphene porous network anchored with uniform Sn nanoparticles (20–40 nm) in the Sn@N-G composite is observed. The element mappings in Figure 2e–h demonstrate that those nanoparticles contain Sn, N, and C elements, which are uniformly distributed in the composite, demonstrating the successful synthesis of the Sn@N-G composite. The novel fold structure of graphene wrapping can prevent the Sn nanoparticles from creeping down effectively and provide a buffer space for Sn swelling during repeated cycling. Moreover, the addition of melamine provides a plentiful nitrogen source for the Sn/N-G composite. The nitrogen atoms were successfully doped into graphene layers during thermal treatment, as shown in Figure 2h. The Sn content (~42 wt%) of the Sn@N-G composite was determined by Thermogravimetric Analysis (TGA) experiment (Figure S3).

**Figure 3:**
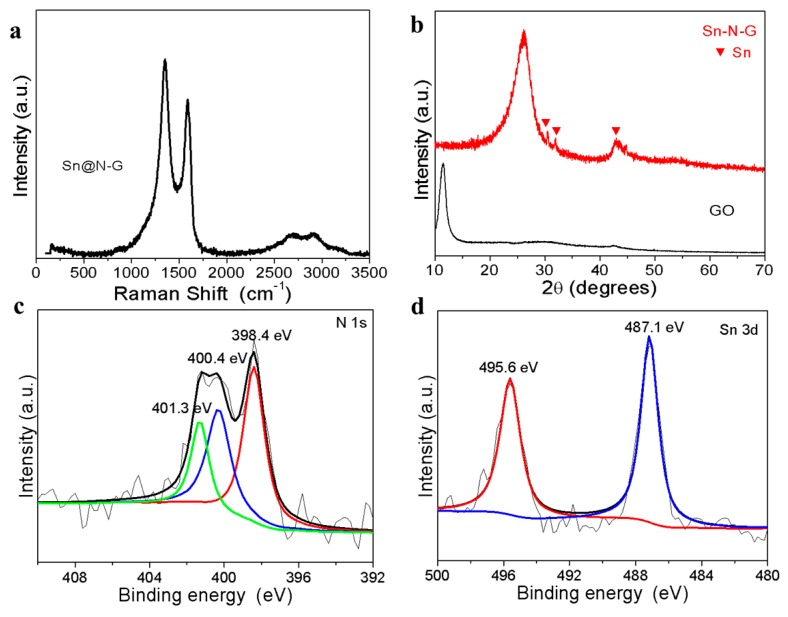
(**a**) Raman spectrum of Sn@N-G composite. (**b**) X-ray photoelectron spectroscopy (XRD) patterns of grapheme oxide (GO) and Sn@N-G, (**c**) X-ray photoelectron spectroscopy (XPS) spectrum of N1s of Sn@N-G composite. (**d**) XPS spectrum of Sn 3D Sn@N-G composite.

**Figure 4:**
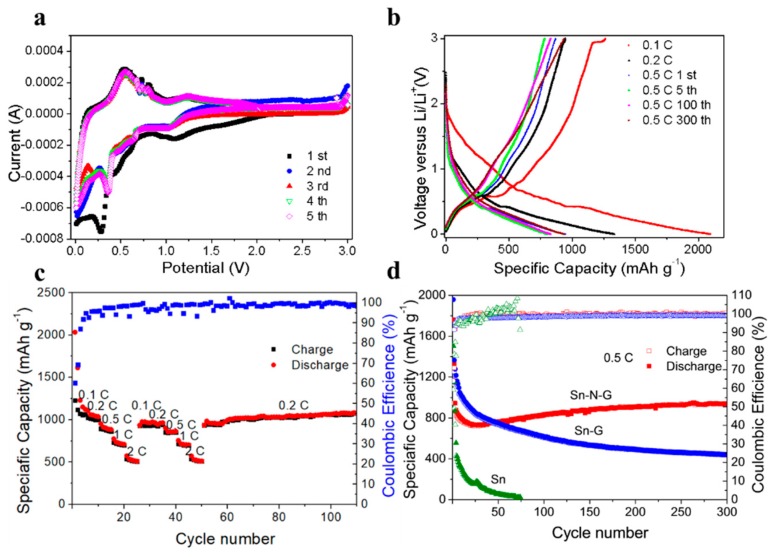
(**a**) Charge-discharge curves of the Sn@N-G anode at different current densities. (**b**) Cyclic voltammetry (CV) curves of the Sn@N-G anode at a scan rate of 0.1 mV/s. (**c**) Rate capability of the Sn@N-G anode. (**d**) Cycling performance comparison of Sn, Sn-graphene, and Sn@N-G electrodes.

In order to further obtain better information on the as-prepared samples, the Raman spectroscopy was collected to evaluate the characteristics and quality of the graphene in the composite, as shown in Figure 3a. The D band at 1350 cm^−1^, G band at 1580 cm^−1^, and 2D band at 2700 cm^−1^ of graphene are clearly observed in the spectrum, which corresponds to the first-order zone boundary phonon mode associated with edges, other defects, or disordered carbon, the radial C–C stretching mode of ordered sp^2^ carbon, and the second-order zone boundary phonon mode for graphene, respectively. The relative intensity of the D band to the G band (I_D_/I_G_) is 1.24 (Figure 3a), which is much higher than that of pure graphene shown in previous works. The high ratio of I_D_/I_G_ indicates the high degree of disorder from the graphitic structure, which may originate from the N doping of the graphene sheets. The XRD pattern in Figure 3b shows that there is no diffraction peak of Sn@N-G at 12° and a broad peak at 26° appeared, which indicates that GO was reduced after addition of SnCl_2_ and subsequent heating treatment. As a comparison, the XRD pattern of GO has a characteristic peak located at 12°. Besides, several characteristic peaks belonging to the Sn crystal structure (JCPDS No. 04-0673) appear at 30.6°, 32°, and 44.9°, indicating Sn in the as-prepared sample exists mainly in the form of Sn metal. The Sn@N-G composite was also analyzed using XPS (Figure 3c,d and Figure S4). Figure 3c displays the high-resolution N 1s spectrum of Sn@N-G composite. The binding energy centered at 398.4, 400.4, and 401.3 eV is attributed to pyridinic-N, pyrrolic-N, and graphitic-N, respectively [28]. The nitrogen content of Sn/Nitrogen-doped graphene nanocomposites is calculated to be approximately 4.2 wt% (Table S1). The high-resolution Sn 3D spectra are shown in Figure 4d. The Sn 3D spectra presented two peaks at 487.1 (Sn 3d 5/2) and 495.6 eV (Sn 3d 3/2), which are higher than the corresponding binding energy of Sn^0^, indicating the existence of Sn^4+^ on the surface of Sn nanoparticles, which is due to the oxidation of the highly active Sn metal surface, and has been widely observed in reported Sn and carbon (Sn@carbon) compositions [29,30,31].

To investigate the lithium storage performance of the as-prepared Sn@N-G composite, CR2032 type lithium-ion half cells were constructed for the electrochemical tests. The cyclic voltammograms (CVs) of the first three cycles of Sn@N-G anodes are shown in Figure 4a. A reduction peak at 1.1 V (vs. Li^+^/Li) is observed in the first cathodic scan and the intensity of this peak decreases in the following cycles, which corresponds to the irreversible reactions related to the formation of SEI films. The broad reduction peaks near 0.5 V is associated with the alloying reaction and formation the Li-Sn alloy based on the reaction of Sn + *x*Li^+^ + e^−^ → Li*_x_*Sn, which is in accordance with previous reports of Sn-based anodes [32]. For the anodic scanning, oxidation peaks between 0.5 V and 1.0 V (vs. Li^+^/Li) are related to the desorption of Li^+^ from the graphene surface and the Li_x_Sn de-alloying reaction. In the following cycle, same cathodic and anodic peaks appeared as in the first cycle, indicating the high Li-storage reversibility of the Sn@N-G anode. Importantly, after the third cycle, the intensity of the oxidation and reduction peaks is almost identical, suggesting a stable SEI formed after the first 3 cycle. In the CV curves, the oxidization and reduction peaks are consistent with the charge–discharge plateaus in Figure 4a.

Figure 4b shows the charge–discharge curves of the Sn@N-G anode at different current densities with a voltage range between 0.01 and 3 V (vs. Li^+^/Li). At a current density of 0.1C, the as-prepared anode delivers an initial discharge capacity of 2098 mAhg^−1^, which is higher than the theoretical specific capacity of Sn. The high initial discharge capacity may originate from the N-doping sites, which serve as reservoirs for Li^+^ storage, and the solid electrolyte interface (SEI) formation. In addition, a long plateau near 0.5 V (vs. Li^+^/Li) can be observed and the corresponding charge plateau also appeared near 0.5 V, which indicates a small polarization effect during the charge–discharge process. The charge–discharge behaviors of the Sn@N-G anode at current densities of 0.2C and 0.5C are displayed in Figure 4b. High specific capacities of 1350 and 950 mAh g^−1^ are obtained at 0.2C and 0.5C. The charge–discharge curves of different cycles at 0.5C are also shown in Figure 4b. Even after 300 cycles a similar curve can be observed, demonstrating the stable cycling performance of the Sn@N-G composite.

The rate capability and cycling performances of the Sn@N-G anode are shown in Figure 4c. The Sn@N-G anode delivers specific capacities of 1226, 1027, 894, 729, and 535 mAh g^−1^, respectively, at current densities of 0.1, 0.2, 0.5, 1, and 2C. As the current density turns back to 0.1 C, a high capacity of 933 mAh g^−1^ (~76% of the initial one) is recovered. This superior rate capability is ascribed to the good electronic conductivity of the Sn@N-G composite. The 3D graphene conductive network facilitates the Li^+^ and electron transportation effectively, and N-doping further improves the electronic conductivity of the graphene sheets. The cycling stability of Sn@N-G, Sn-graphene, and pure Sn anode is compared in Figure 4d. The pure Sn anode exhibits a very poor cycling performance with fast capacity fading. Correspondingly, the Coulombic efficiency (CE) fluctuates drastically, suggesting unstable SEI during cycling due to the large volume expansion of Sn. After compositing Sn with graphene, the cycling performance is improved obviously, indicating that graphene wrapping accommodates the volume change effectively and prevents the direct contact of Sn with electrolytes. Meanwhile, the CE of Sn@graphene becomes more stable during cycling. However, the specific capacity of the Sn-graphene composite electrode fades quickly in the first 50 cycles. In contrast, the Sn@N-G composite anode has a very stable cycling performance at a current density of 0.5C. The capacity is maintained at 939 mAhg^−1^ after 300 cycles, implying good cycling stability for the Sn@N-G anode (Figure S5). TEM images showed that its remaining structure was almost unchanged. After 100 cycles at 0.2C and 500 cycles at 1C, the capacity was maintained at 1093.9 mAhg^−1^ and 764.8 mAhg^−1^, respectively, as shown in Figures S6 and S7. The excellent electrochemical performance of the Sn@N-G composite compares favorably with that of previously reported Sn anodes for LIBs (Table S2).

Interestingly, the capacity of Sn@N-G shows a capacity increasing during long-term cycling, which may be attributed to the improved electrolyte wetting upon cycling and reconstruction of Sn nanoparticles during repeated cycling processes, similar to other conversion reaction-type anodes reported previously [33,34]. Compared with Sn@graphene, the higher specific capacity of Sn@N-G originates from N-doping, which creates large amounts of active sites for Li^+^ storage, as reported previously [32]. Overall, the superior rate capability and cycling performance of the Sn@N-G composite electrode should be attributed to the 3D N-doping graphene conductive network that accommodates the large volume change of Sn during charge–discharge and improves the ionic and electronic conductivity of the electrode, as well as provides rich active sites for additional Li^+^ storage. 

## 4. Conclusions

In summary, a Sn@N-G composite anode has been successfully fabricated via a facile method. The Sn nanoparticles are uniformly wrapped by 3D graphene layers, which can accommodate the large volume change of Sn particles during the charge–discharge process and provide a conductive network. Moreover, the introduction of N element creates more active sites and improves the electronic conductivity of graphene. As a result, the capacity, rate capability, as well as cycling performance of the Sn@N-G anode is substantially improved. It delivers a high capacity of 535 mAh g^−1^ at a current density of 2C, and stable cycling performance of 300 cycles at 0.5C. The superb electrochemical performance of Sn@N-G demonstrates that the facile method is an effective way to synthesize N-doped carbon composite electrodes, which should provide a new solution for developing high-performance materials for batteries applications.

## Supplementary Materials

The following are available online at https://www.mdpi.com/2079-4991/9/8/1084/s1, Figure S1: SEM image of Sn@N-G precursor (melamine); Figure S2: Optical images of Sn@G and Sn@N-G composite paticles; Figure S3: TG resutlt of Sn/N-G obtain in air; Figure S4: XPS spectrum of C1s, O1s and survey XPS spectrum of Sn@N-G composite; Figure S5: TEM image of Sn@N-G composite after 100 cycles at 0.5C; Figure S6: Cycling performance of Sn@N-G electrodes at 0.2C; Figure S7: Cycling performance of Sn@N-G electrodes at 1C; Table S1: The contents of carbon, nitrogen, oxygen, and tin in the Sn/N-G composite; Table S2: Comparison of electrochemical performances of the Sn@N-G electrodes with previously reported Sn-based electrodes.
