# Supplementary material for: Facile Synthesis of Sn/Nitrogen-Doped Reduced Graphene Oxide Nanocomposites with Superb Lithium Storage Properties
Source: Nanomaterials (Basel). 2019 Jul 28;9(8):1084. doi: 10.3390/nano9081084 (PMC6723252; doi:10.3390/nano9081084)
Supplement: Supplementary file 1 [file nanomaterials-09-01084-s001.pdf]

Supporting Information

Facile Synthesis of Sn/Nitrogen-Doped Reduced Graphene Oxide Nanocomposites with Superb Lithium Storage Properties

Quan Sun[1], Ying Huang[1], Shi Wu[1], Zhonghui Gao[1], Hang Liu [2], Pei Hu[2,3,*] and Long Qie[1,*]

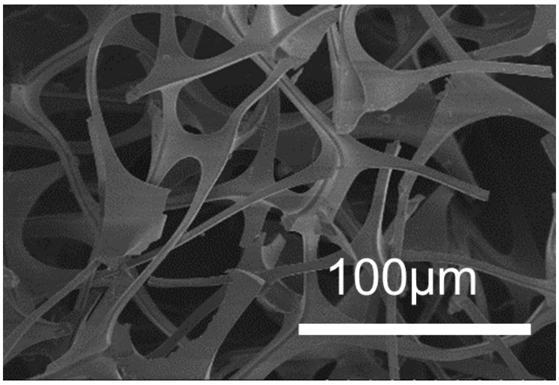

Figure S1. SEM image of Sn@N-G precursor(melamine)

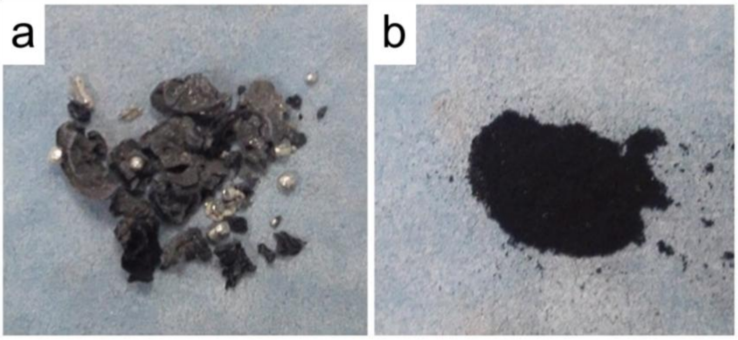

Figure S2. Optical images of Sn@G and Sn@N-G composite paticles

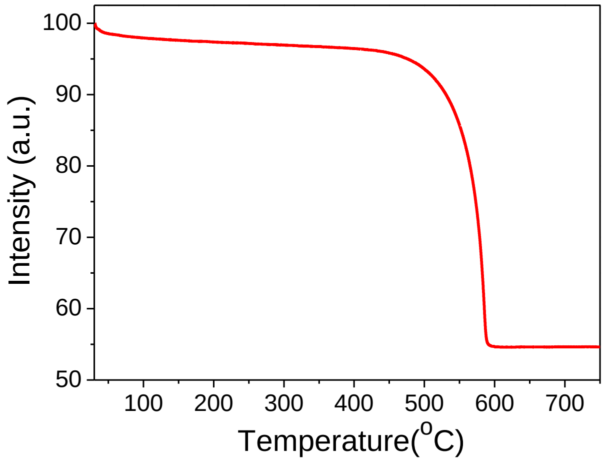

Figure S3. TG resutlt of Sn/N-G obtain in air.

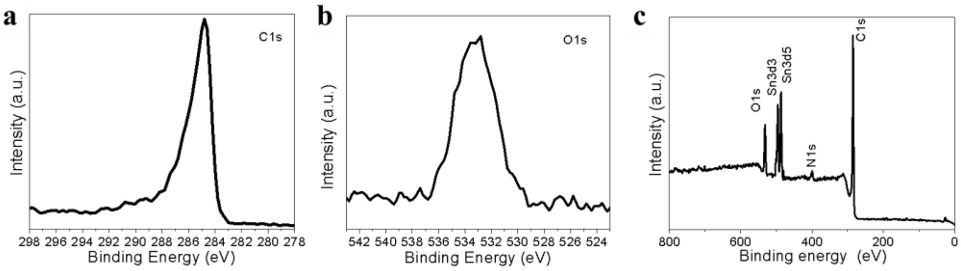

Figure S4. XPS spectrum of C1s, O1s and survey XPS spectrum of Sn@N-G composite.

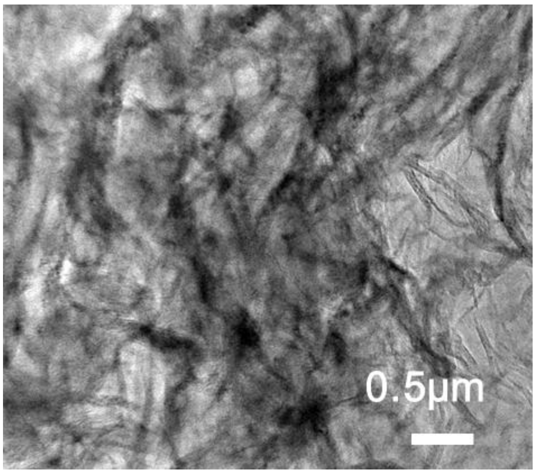

Figure S5. TEM image of Sn@N-G composite after 100 cycles at 0.5C

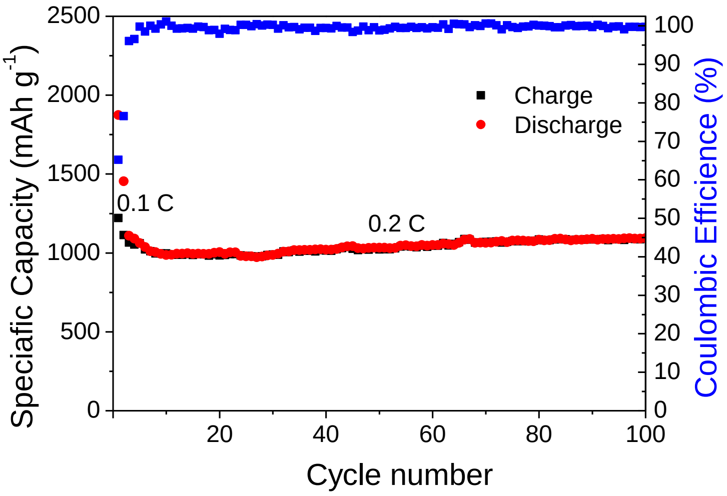

Figure S6. Cycling performance of Sn@N-G electrodes at 0.2C.

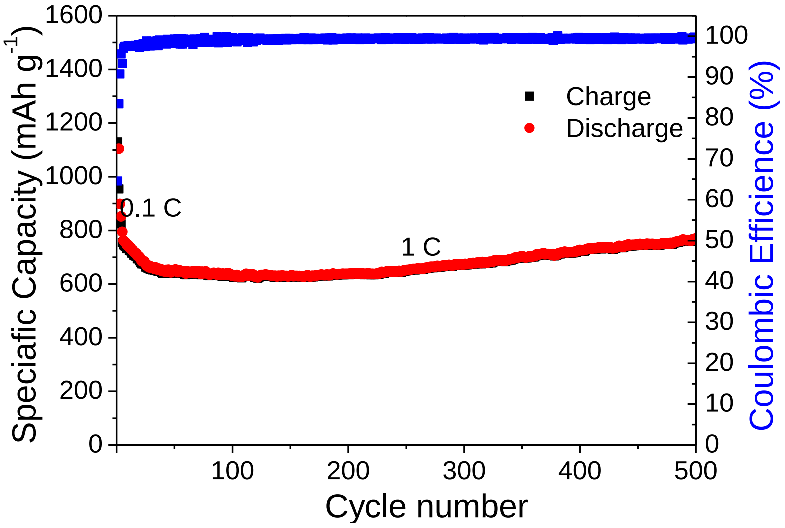

Figure S7. Cycling performance of Sn@N-G electrodes at1C.

Table S1 The contents of carbon, nitrogen, oxygen, and tin in the Sn/N-G composite.

Sample	C (wt%)	N (wt%)	O (wt%)	Sn (wt%)
Sn/N-G	44.4	4.2 %	9.8	41.6 %

Table S2 Comparison of electrochemical performances of the Sn@N-G electrodes with previously reported Sn-based electrodes.

**Electrode materials**	**Synthetic method**	**Cycling stability (C/A/n)**	**Ref.**
Sn/C-PANI nanocomposite	hydrogen thermal reduction	650/100/300	S1
Sn/N-doped carbon nanospheres nanocomposites	arc-discharge	520.4/500/1000	S2
Nano-Sn/C composites	aerosol spray pyrolysis	710/200/130	S3
Sn/C composite	chemical vapor deposition + chemical transformation	626.4/600/200	S4
Sn@C/graphenenanocomposite	hydrothermal	662/100/100	S5
core/shell-structured Sn/onion-like carbon nanocapsules	arc-discharge	585/100/100	S6
Sn–C nanostructured composite	gel + calcination	500/1000/200	S7
Sn@Graphene	chemical vapor deposition	682/2000/1000	S8
Sn nanoparticles decorated 3D foothill-like graphene	microwave plasma enhanced chemical vapor deposition	794/293/400	S9
Sn-based nanoparticles on graphene	chemical transformation + heat treatment	565.1/200/50	S10
Sn/nitrogen-doped carbon composite	hydrolyze + pyrolyze	660/200/200	S11
Pitaya-like Sn@C nanocomposites	spray pyrolysis	910/200/180	S12
Sn-carbon nanotube nanocapsules	Arc discharge	612/100/10	S13
C/Sn and C/SnO/Sn composites	gel + heat treatment	939/500/300	S14
Sn@N-G composites			This work

C/A/n means the capacity of C (mAh g^{-1}) remained after n cycles at the certain currentdensity of A (mA g^{-1}).

References

S1 X. Chang, T. Wang, Z. Liu, X. Zheng, J. Zheng, X. Li, Ultrafine Sn Nanocrystals in a Hierarchically Porous N-doped Carbon for Lithium Ion Batteries, Nano Res. 2017, 10, 1950-1958.

S2 S. Liang, X. Zhu, P. Lian, W. Yang, H. Wang, Superior cycle performance of Sn@C/graphene nanocomposite as an anode material for lithium-ion batteries. J. Solid State Chem., 2011, 184, 1400-1404.

S3 Y. Xu, Q. Liu, Y. Zhu, Y. Liu, A. Langrock, M. R. Zachariah, and C. Wang, Uniform Nano-Sn/C Composite Anodes for Lithium Ion Batteries. Nano Lett., 2013, 13, 470-474.

S4 X. Huang, S. Cui, J. Chang, P. B. Hallac, C. R. Fell, Y. Luo, B. Metz, J.i Jiang, P. T. Hurley, and J. Chen, A Hierarchical Tin/Carbon Composite as an Anode for Lithium-Ion Batteries with a Long Cycle Life . Angew. Chem. 2015, 127, 1510–1513.

S5 S. Liang, X. Zhu, P. Lian, W. Yang, HaihuiWang, Superior cycle performance of Sn@C/grapheme nanocomposite as ananode material for lithium-ion batteries. J. Solid State Chem., 2011, 184, 1400-1404.

S6 C. Cui, X. Liu, N. Wu, Y. Sun, Facile synthesis of core/shell-structured Sn/onion-like carbon nanocapsules as High performance anode material for lithium-ion batteries, Mater. Lett., 2015, 143 , 35-37.

S7 G. Derrien, J. Hassoun, S. Panero, and B. Scrosati, Nanostructured Sn–C Composite as an Advanced Anode Material in High-Performance Lithium-Ion Batteries. Adv. Mater., 2007, 19, 2336–2340.

S8 J. Qin, C. He, N. Zhao, Z. Wang, C. Shi, E. –Z. Liu, and J. Li, Graphene Networks Anchored with Sn@Graphene as Lithium Ion Battery Anode. ACS Nano, 2014, 8, 1728-1738.

S9 C. Wang, Y. Li, Y. Chui, Q. Wu, X. Chen and W. Zhang, Three-dimensional Sn-graphene anode for high-performance lithium-ion battery. Nanoscale, 2013, 5, 10599-10604.

S10 Z. Wen, S. Cui, H. Kim, S. Mao, K. Yu, G. Lu, H. Pu, O. Mao and J. Chen, Binding Sn-based nanoparticles on graphene as the anode of rechargeable lithium-ion batteries. J. Mater. Chem., 2012, 22, 3300-3306.

S11 D. Youn, A. Heller, and C. B. Mullins, Simple synthesis of nanostructured Sn/nitrogen-doped carbon composite using nitrilotriacetic acid as lithium ion battery anode. Chem. Mater., 2016, 28, 1343−1347.

S12 N. Zhang, Q. Zhao, X. Han, J. Yang and J. Chen, Pitaya-like Sn@C nanocomposites as high-rate and long-life anode for lithium-ion batteries. Nanoscale, 2014, 6, 2827–2832.

S13 C.-j. Liu, H. Huang, G.-z. Cao, F.-h. Xue, R.A.P. Camacho, X.-l. Dong, Enhanced Electrochemical Stability of Sn-Carbon Nanotube Nanocapsules as Lithium-Ion Battery Anode, Electrochimica Acta, 2014, 144, 376-382.

S14 Peng Liu, Wu Lei, Xifeng Xia, and Qingli Hao, Controllable fabrication of C/Sn and C/SnO/Sn composites as anode materials for high-performance lithium-ion batteries. Chem. Eng. J., 2017, 330, 1035–1043.
